# CRIF1 attenuates doxorubicin-mediated mitochondrial dysfunction and myocardial senescence via regulating PXDN

**DOI:** 10.18632/aging.205664

**Published:** 2024-03-15

**Authors:** Lina Zhou, Guilan Zhai, Ge Tian

**Affiliations:** 1 Department of Geriatrics, The First Affiliated Hospital of Jinzhou Medical University, Jinzhou 121000, Liaoning, China; 2 Department of Cardiology, The First Affiliated Hospital of Jinzhou Medical University, Jinzhou 121000, Liaoning, China

**Keywords:** myocardial senescence, CRIF1, PXDN, mitochondrial function

## Abstract

Background: CR6-interacting factor 1 (CRIF1), a multifunctional protein that affects mitochondrial function and cell senescence, plays a regulatory role in heart-related diseases. However, whether CRIF1 participates in myocardial senescence by regulating mitochondrial function remains unclear.

Methods: Doxorubicin (DOX)-induced C57BL/6 mice to construct mouse myocardial senescence model, and the myocardial function indicators including lactate dehydrogenase (LDH) and Creatine kinase isoform MB (CK-MB) were assessed. The expression of CRIF1 was detected by western blot. Myocardial pathological changes were examined by transthoracic echocardiography and haematoxylin and eosin (H&E) staining. Cell senescence was detected by SA-β-gal staining. JC-1 staining was used to detect mitochondrial membrane potential. Biochemical kits were used to examine oxidative stress-related factors. Additionally, AC16 cardiomyocytes were treated with DOX to mimic the cellular senescence model *in vitro*. Cell activity was detected by cell counting kit-8 (CCK-8) assay. Co-immunoprecipitation (CO-IP) was used to verify the relationship between CRIF1 and peroxidasin (PXDN).

Results: The CRIF1 expression was significantly decreased in DOX-induced senescent mice and AC16 cells. Overexpression of CRIF1 significantly ameliorated DOX-induced myocardial dysfunction and myocardial senescence. Additionally, CRIF1 overexpression attenuated DOX-induced oxidative stress and myocardial mitochondrial dysfunction. Consistently, CRIF1 overexpression also inhibited DOX-induced oxidative stress and senescence in AC16 cells. Moreover, CRIF1 was verified to bind to PXDN and inhibited PXDN expression. The inhibitory effects of CRIF1 overexpression on DOX-induced oxidative stress and senescence in AC16 cells were partly abolished by PXDN expression.

Conclusions: CRIF1 plays a protective role against DOX-caused mitochondrial dysfunction and myocardial senescence partly through downregulating PXDN.

## INTRODUCTION

Cardiovascular disease is the leading cause of death, accounting for 31% of all deaths worldwide [1]. Aging is a major risk factor for cardiovascular diseases [2]. Cellular senescence, classically defined as stable cell cycle arrest, is a hallmark of aging [3]. Studies have found that there are a large number of senescent cells in the elderly and diseased hearts, which eventually leads to pathological changes in heart structure and function, such as left ventricular hypertrophy and decreased diastolic function, extracellular matrix remodeling, increased myocardial fibrosis and conduction block [4, 5]. Currently, the molecular mechanisms responsible for cardiac senescence remains largely unclear, and thus there are no effective options to prevent cardiac senescence.

CR6-interacting factor 1 (CRIF1) is a multifunctional protein that affects mitochondrial oxidative respiration and participates in the negative regulation of the cell cycle and DNA damage response [6]. CRIF1 is widely distributed in a variety of organs, including the brain, liver, intestine, and heart [7]. A study has shown that the loss of CRIF1 impairs mitochondrial oxidation function and is associated with excessive oxidative stress in different cell lines [8]. In addition, CRIF1 can interact with the large subunits of mitochondrial ribosomes, and CRIF1 deficiency can lead to increased production of mitochondrial reactive oxygen species (ROS), resulting in endothelial dysfunction [9]. However, mitochondrial dysfunction is the core of the senescence theory, and senescence is related to the decline of mitochondrial function and the reduction of mitochondrial ATP synthesis [10]. Therefore, it is possible that CRIF1 regulates mitochondrial function in myocardial senescence, suggesting the potential regulatory mechanism of CRIF1 in myocardial senescence. Other studies have shown that specific absence of CRIF1 in endothelial cell leads to heart defects [11], and that CRIP1 deficiency can induce myocardial hypertrophy [12]. These results indicate that CRIF1 can participate in the occurrence of cardiovascular and cerebrovascular circulatory system-related diseases. However, whether CRIF1 can participate in myocardial senescence by regulating mitochondrial function is still unknown.

In our article, we explored the role and regulation of CRIF1 in myocardial senescence and its regulatory mechanism related to mitochondrial function through *in vivo* and *in vitro* experiments. Our paper might provide a reliable theoretical basis for the clinical treatment of myocardial senescence.

## MATERIALS AND METHODS

### Animals

Total 48 C57BL/6 male mice (8–10 weeks, 23–28 g) obtained from Beijing HFK Bioscience (Beijing, China) were subjected to an adaptive feeding for 1 week before the experiment commenced. To establish a cardiomyocyte senescence model, mice were intraperitoneally injected with doxorubicin (DOX; HY-15142A, MedChemExpress, USA) via a total of six injections, each containing 2.5 mg/kg DOX, which lasted two weeks [13]. Mice in control group were administered with an equivalent volume of normal saline. Viral vectors including negative control (AAV-NC) and AAV-CRIF1 were delivered into mice by tail vein injection 4 weeks before DOX administration, at a concentration of 1 × 10^11^ viral genome per mouse. Animal experiments were carried out in compliance with the Guidelines for the Care and Use of Laboratory Animals published by the United States National Institutes of Health (NIH Publication, revised 2011) and approved by the Animal Care and Use Committee of The First Affiliated Hospital of Jinzhou Medical University.

### Measurement of lactate dehydrogenase (LDH) activity and creatine kinase isoform MB (CK-MB) activity

After mouse serum was harvested, the supernatant was obtained by centrifugation (350 g, 10 min, 4° C) and the LDH activity was detected using the LDH kit (Jiancheng, Nanjing, China) at 530 nm using a microplate reader (Benchmark; Bio-Rad Laboratories, Inc., Berkeley, CA, USA). CK-MB was quantitated by using a commercial colorimetric assay kit (Pars Azmoon, Tehran, Iran) as per the instructions stipulated by the manufacturer.

### Real time-quantitative PCR (RT-qPCR)

Total RNA was isolated from cardiac tissues and cells using the Trizol reagent (Thermo Fisher Scientific, Inc., Waltham, MA, USA). Complementary DNA (cDNA) was generated from total RNA using HiScript^TM^ First-strand cDNA Synthesis Kit (Vazyme, China). Relative RNA levels were detected by RT-PCR using the Prism 7000 Sequence Detection System (Applied Biosystems, Foster City, CA, USA) with the Super Script III Platinum SYBR Green One-Step qRT-PCR Kit (Invitrogen, Carlsbad, CA, USA). The mRNA levels were calculated using the 2^−ΔΔCq^ method.

### Western blot analysis

Proteins were extracted from cardiac tissues and cells with RIPA lysis buffer. After centrifugation, the supernatant was collected as the total protein. Each 20 μg protein was separated by electrophoresis using 10% SDS-PAGE and then transferred to PVDF membranes (Bio-Rad Laboratories, Inc.). The membranes were blocked with 5% BSA in TBST for 1 h at room temperature, followed by incubation with primary antibodies including anti-CRIF1 (dilution 1:2,000, ab226244, Abcam, UK), anti-p27 (dilution 1:5,000, ab32034, Abcam), anti-p21 (dilution 1:1,000, ab109199, Abcam), anti-p16 (dilution 1:1,000, sc-1661, Santa Cruz Biotechnology, USA), anti-peroxidasin (PXDN; dilution 1:1,000, orb544131, Biorbyt, UK), anti-Flag (dilution 1:1,000, ab205606, Abcam), anti-Myc (dilution 1:1,000, ab9106, Abcam) and anti-GAPDH (dilution 1:2,500, ab9485, Abcam) at 4° C overnight. Thereafter, after incubation with goat anti-rabbit horseradish peroxidase-conjugated secondary antibody (dilution 1:2,000, ab6721, Abcam) for 2 h at room temperature, membranes were detected by an enhanced chemiluminescence (ECL) detection system (Beyotime Institute of Biotechnology, Shanghai, China) and the density of the bands was determined using ImageJ software (NIH, Bethesda, MD, USA).

### Determination of cardiac function

Cardiac function was assessed using a Vevo 3100 high-resolution imaging system (MS-550D; VisualSonics, Toronto, Canada) equipped with a 30 MHz sensor with transthoracic echocardiography. Left ventricular ejection fraction (LVEF) and left ventricular fractional shortening (LVFS) were calculated by computer algorithms.

### Haematoxylin and eosin (H&E) staining

Cardiac tissues were fixed in formalin at 4° C overnight, embedded in paraffin and cut into 4-μM sections. H&E staining of the sections was then performed and the stained sections were visualized under a light microscope (Olympus, Tokyo, Japan).

### Senescence-associated β-galactosidase (SA-β-gal) staining

The SA-β-gal staining kit (Beyotime Institute of Biotechnology) was used to assess cellular senescence in accordance with the manufacturer’s instructions. In brief, cells and frozen tissue sections were washed with PBS, and then fixed with fixative solution for 10-15 min, followed by incubation with the staining solution at 37° C overnight. Images were observed under a microscope (Olympus, Tokyo, Japan).

### JC-1 staining

JC-1 staining kit (cat. no. C2006; Beyotime Institute of Biotechnology) was used to detect the mitochondrial membrane potential (MMP). Cells were stained by JC-1 (10 μg/mL) at 37° C for 20 min. MMP was visualized by a fluorescence microscopy (Olympus, Tokyo, Japan). Red fluorescence represented aggregates. Green fluorescence represented the monomeric form of JC-1.

### Measurement of malondialdehyde (MDA), glutathione (GSH), catalase (CAT) and ROS

The expression levels of MDA (A003-1-2, Nanjing Jiancheng, Nanjing, China), GSH (A006-1-1, Nanjing Jiancheng, Nanjing, China) and CAT (A007-2-1, Nanjing Jiancheng, Nanjing, China) in myocardial tissue homogenate or cell supernatant were detected according to the relevant kit instructions. The intracellular ROS level was determined using the commercial kits (Sigma-Aldrich, St. Louis, MO, USA) based on the turn out of the 2’7’-dichlorofluorescin diacetate (DCF-DA) into highly fluorescent 2’,7’-dichlorofluorescein (DCF) in line with the manufacturer’s instructions. The fluorescence intensity was measured by a fluorescence microscopy (Olympus, Tokyo, Japan).

### Cell culture

Human cardiomyocyte AC16 cells (Cat. no. BNCC339980) and 293T cells (Cat. no. BNCC353535) were purchased from BeNa Culture Collection (Zhengzhou, China). AC16 cells were maintained in DMEM medium (Invitrogen, Carlsbad, CA, USA) with 10% fetal bovine serum (FBS, Gibco, Waltham, MA, USA) at 37° C at an atmosphere of 5% CO_2_. DOX at different doses of 1.25 μM, 2.5 μM, and 5 μM was used to treat AC16 cells for 24 h [14].

### Cell counting kit-8 assay

Cell Counting Kit-8 (CCK-8) assay was used to measure cell proliferation. AC16 cells were seeded into a 96-well plate at the density of 5×10^3^ cell/well at 37° C with 5% CO_2_. After cells were subjected to indicated treatment, 10 μL of CCK-8 reagent (Dojindo Laboratories, Kumamoto, Japan) was added to each well for 2 h of incubation. A microplate reader (Bio–Rad, Berkeley, CA, USA) was used to measure the absorbance at 450 nm.

### Cell transfection

PcDNA3.1 vectors containing full-length CRIF1 (Ov-CRIF1) and PXDN (Ov-PXDN) and negative control empty pcDNA3.1 vector (Ov-NC; Shanghai GenePharma, Co., Ltd., Shanghai, China) at the concentration of 10 nM were transfected into AC16 cells using Lipofectamine® 3000 (Invitrogen; Thermo Fisher Scientific, Inc.) at 37° C for 48h, according to the manufacturer’s instructions.

In addition, the flag-tagged CRIF1 (Flag-CRIF1; Shanghai GenePharma, Co., Ltd.) and/or myc-tagged PXDN (myc-PXDN) were transfected into 293T cells using Lipofectamine® 3000 (Invitrogen). Further experiments were performed after 48h.

### Co-immunoprecipitation (CO-IP)

Anti-CRIF1 antibody or anti-IgG antibody was added to protein lysates from DOX-induced AC16 cells. After incubation at 4° C overnight, Protein A/G PLUS-Agarose (Santa Cruz Biotechnology) was added to the samples for an incubation at 4° C for 6 h. The immunoprecipitated protein complex were subjected to SDS–PAGE and examined by western blot to examine PXDN expression.

Moreover, to further confirm the interaction between flag-CRIF1 and myc-PXDN, protein lysate from 293T cells with selective overexpression of flag-CRIF1 and myc-PXDN were prepared, followed by CO-IP assay as aforementioned. The myc-tagged protein or flag-tagged protein in immunoprecipitated protein complex was then examined by western blot.

### Statistical analysis

Data were expressed as means ± standard deviation (SD) by GraphPad Prism 8 (GraphPad, La Jolla, CA, USA). Analysis of variance (one-way) followed by Tukey’s test was used to assess the differences. P<0.05 was considered statistically significant.

### Availability of data and materials

The analyzed datasets generated during the present study are available from the corresponding author on reasonable request.

### Consent for participation and publication

All the authors agree to be published.

## RESULTS

### DOX induced myocardial senescence and decreased expression of CRIF1 in mice myocardial tissues

**Figure 1:**
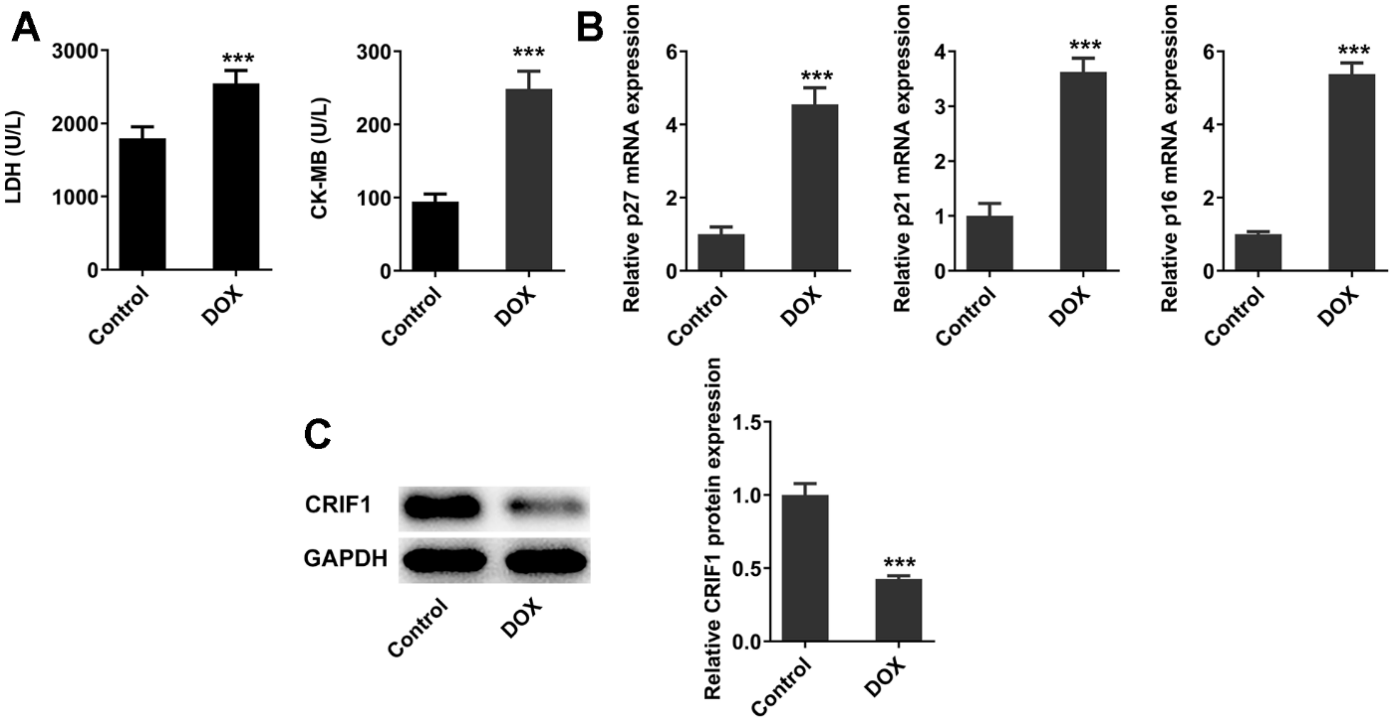
**DOX induced myocardial senescence and decreased expression of CRIF1 in mice myocardial tissues.** (**A**) The expression of myocardial injury-related enzymes LDH and CK-MB in serum was detected by biochemical kits. (**B**) RT-qPCR detected the expression of p27, p21, and p16 in myocardial tissues. (**C**) Western blot detected the expression of CRIF1 in myocardial tissues. ***P<0.001 vs Control.

After DOX induction, the levels of myocardial injury-related enzymes LDH and CK-MB in serum were detected, and the results showed that the serum LDH and CK-MB levels were abnormally elevated after DOX induction compared with those in control group (Figure 1A). RT-qPCR detected the expressions of genes related to cell senescence including p27, p21, and p16 in myocardial tissues, and the results showed that compared with the control group, the expressions of p27, p21, and p16 were significantly increased (Figure 1B), indicating the occurrence of myocardial senescence in DOX-induced mice. In addition, it was revealed from Figure 1C that the protein expression of CRIF1 in myocardial tissues decreased significantly after DOX induction.

### CRIF1 overexpression improved DOX-induced myocardial pathological injury

**Figure 2:**
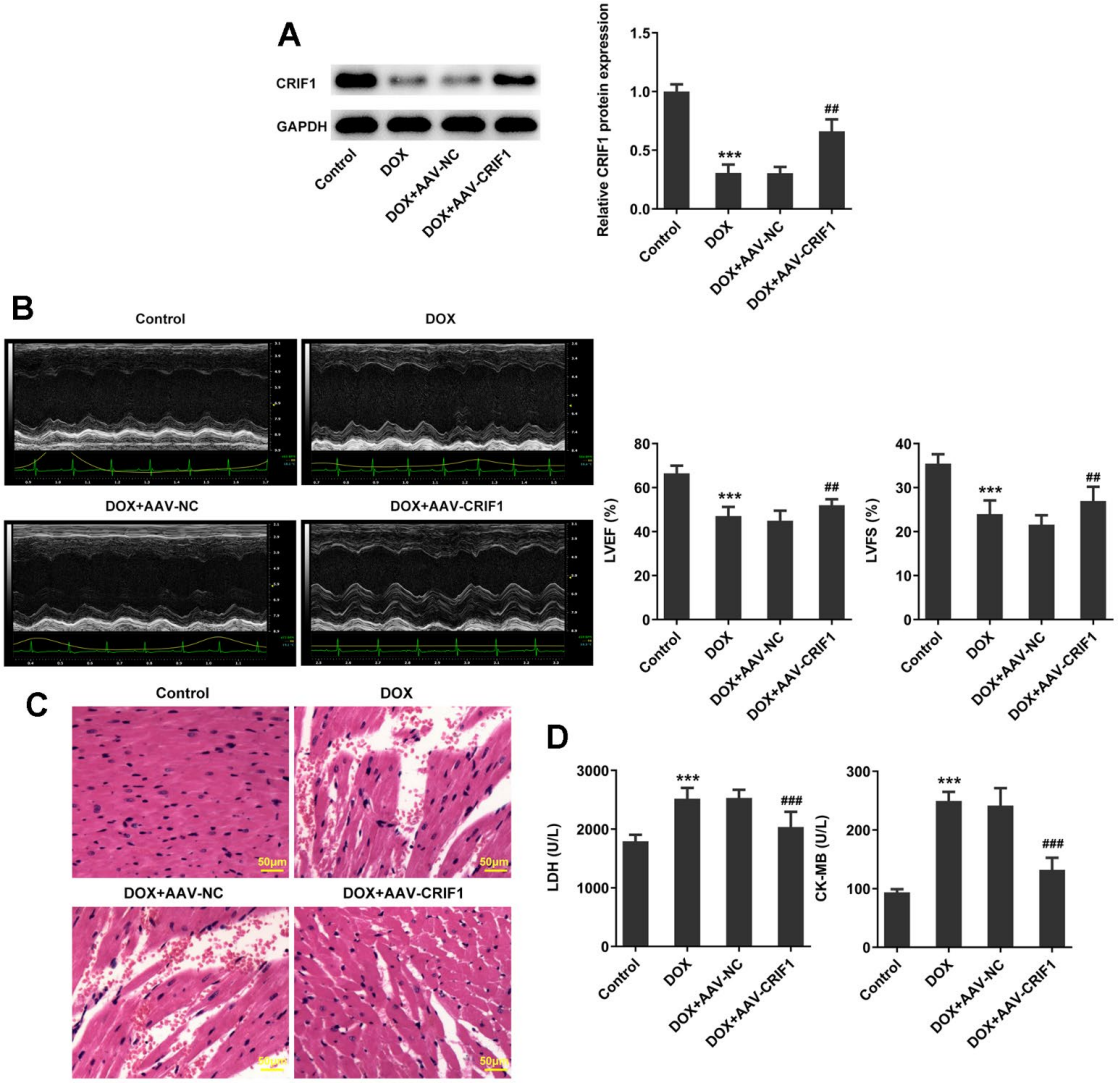
**CRIF1 overexpression improved DOX-induced myocardial pathological injury.** (**A**) AAV-CRIF1 or AAV-NC were delivered into mice prior to DOX administration, and the expression of CRIF1 was detected by western blot. (**B**) Quantitative analysis of cardiac function by echocardiography. (**C**) The pathological changes of myocardium were detected by H&E staining. (**D**) The expressions of LDH and CK-MB in serum were detected by biochemical kits. ***P<0.001 vs Control; ##P<0.01, ###P<0.001 vs DOX+AAV-NC.

To explore the regulatory role of CRIF1 following myocardial senescence, AAV-CRIF1 or AAV-NC were delivered into mice prior to DOX administration. It was revealed that compared with DOX+AAV-NC group, the expression of CRIF1 in DOX+AAV-CRIF1 group was significantly increased (Figure 2A). Quantitative analysis of cardiac function by echocardiography demonstrated that LVEF and LVFS were evidently decreased after DOX induction, while overexpression of CRIF1 could increase LVEF and LVFS (Figure 2B). The pathological changes of myocardium were detected by H&E staining, and the results showed that the myocardium was damaged after DOX induction. However, overexpression of CRIF1 markedly attenuated myocardial tissue damage (Figure 2C). Compared with DOX+AAV-NC group, the expressions of serum LDH and CK-MB in DOX+AAV-CRIF1 group were significantly decreased (Figure 2D).

### CRIF1 overexpression inhibited DOX-induced myocardial senescence

**Figure 3:**
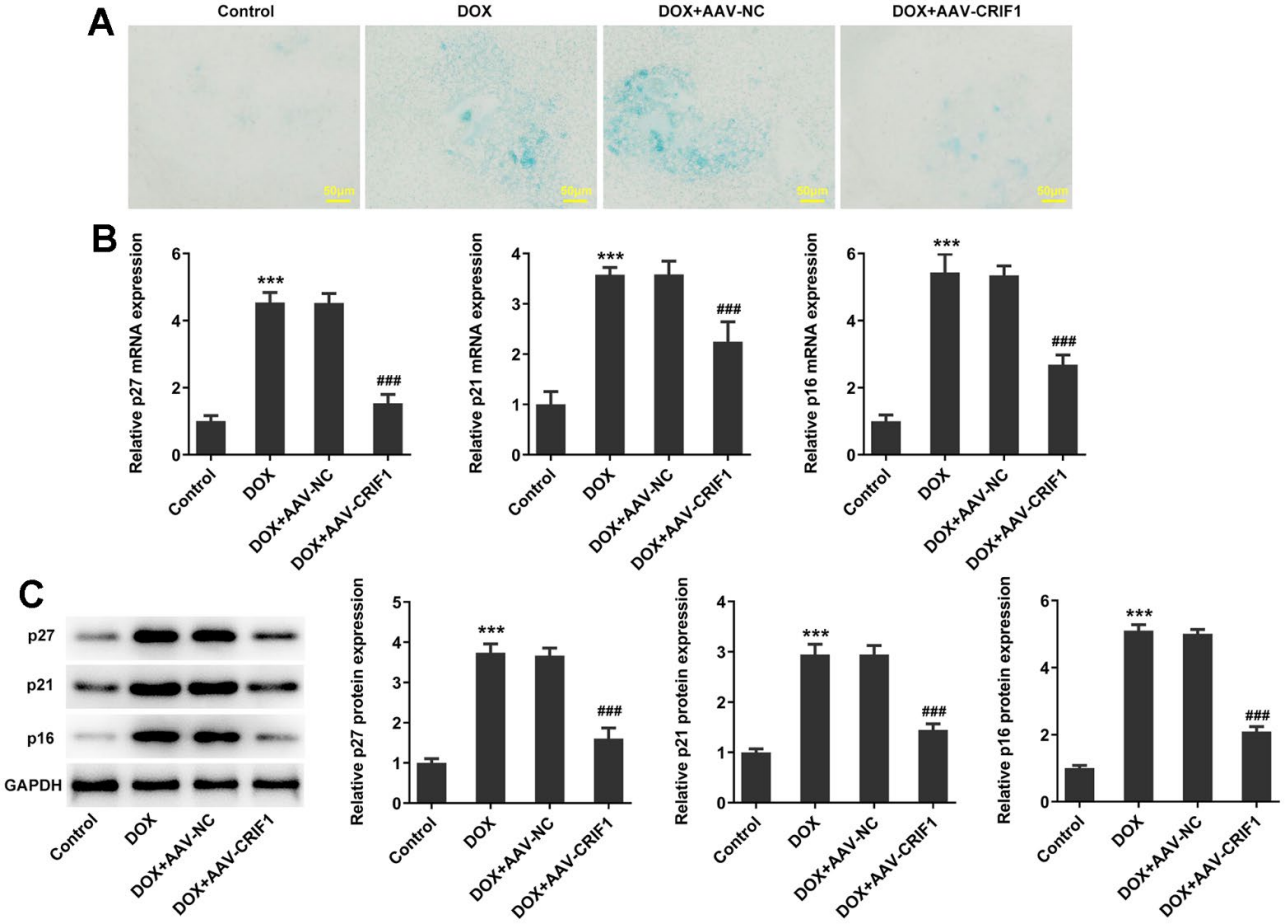
**CRIF1 overexpression inhibited DOX-induced myocardial senescence.** (**A**) The SA-β-gal assay was used to detect myocardial senescence. (**B**, **C**) RT-qPCR and western blot detected the expression of p27, p21, and p16. ***P<0.001 vs Control; ###P<0.001 vs DOX+AAV-NC.

The SA-β-gal staining was used to detect myocardial senescence and the results showed that it exhibited increased levels of SA-β-gal activity in DOX group. After CRIF1 overexpression, SA-β-gal activity decreased (Figure 3A). RT-qPCR and western blot results showed that compared with DOX+AAV-NC group, the expressions of p27, p21 and p16 in DOX+AAV-CRIF1 group were significantly decreased (Figure 3B, 3C).

### CRIF1 overexpression improved DOX-induced myocardial mitochondrial dysfunction

**Figure 4:**
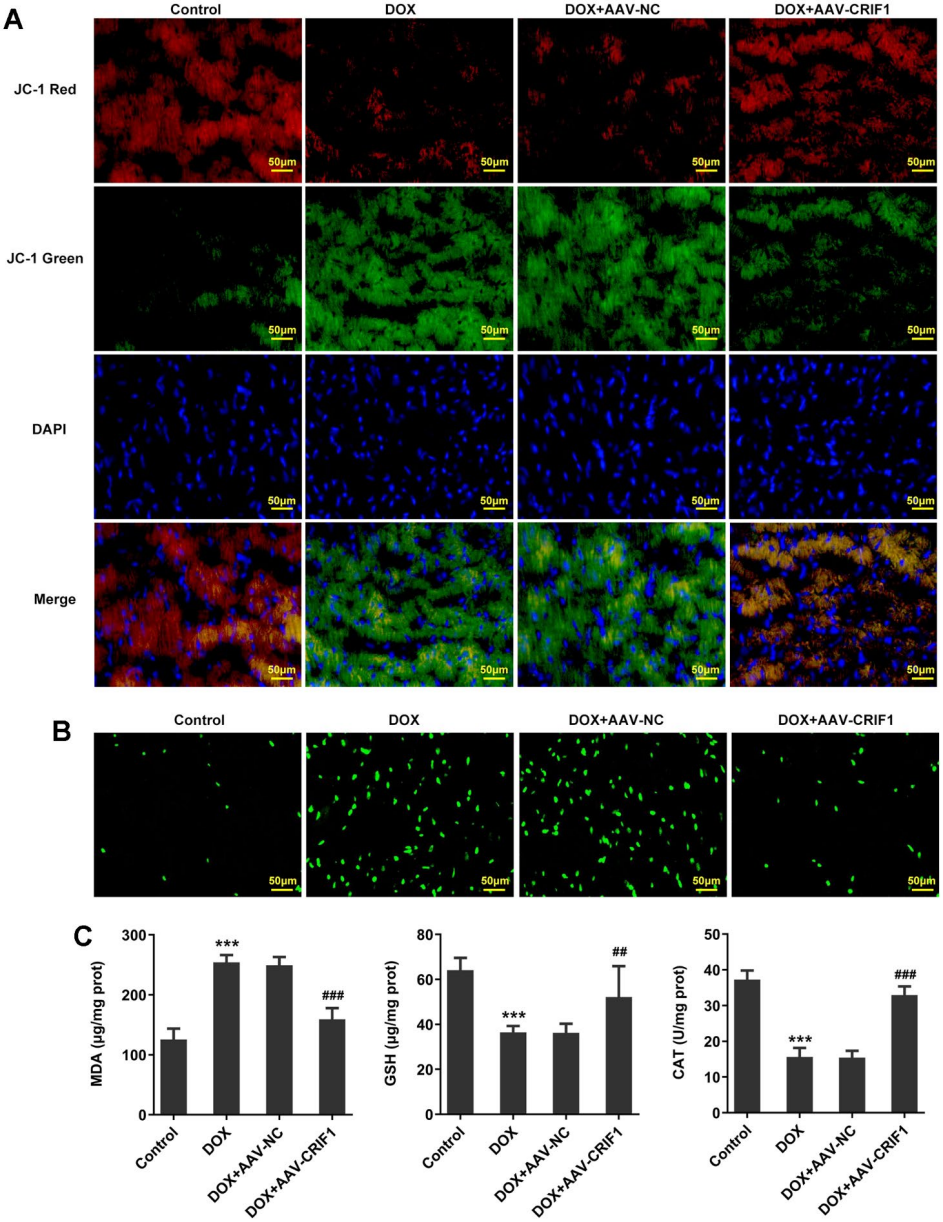
**CRIF1 overexpression alleviated DOX-induced myocardial mitochondrial dysfunction.** (**A**) The changes of MMP were detected by JC-1 staining. (**B**) DCFH-DA staining was used to detect ROS levels. (**C**) The levels of MDA, GSH and CAT in myocardial tissues were detected by biochemical kits. ***P<0.001 vs Control; ##P<0.01, ###P<0.001 vs DOX+AAV-NC.

The changes of MMP were detected by JC-1 staining. We found that JC-1 red decreased and JC-1 green increased after DOX induction, indicating a significant decline in MMP. Compared with DOX+AAV-NC group, MMP was increased in DOX+AAV-CRIF1 group (Figure 4A). DCFH-DA staining was used to detect ROS levels, and the results showed that the ROS level in myocardial tissues was significantly increased after DOX induction, while ROS level was decreased after overexpression of CRIF1 (Figure 4B). The expression levels of MDA, GSH and CAT in myocardial tissues were detected by biochemical kits. We found that MDA expression increased after DOX induction, while GSH and CAT levels decreased, which was partly abolished by overexpression of CRIF1 (Figure 4C).

### The expression of CRIF1 was decreased in DOX-induced AC16 cells

**Figure 5:**
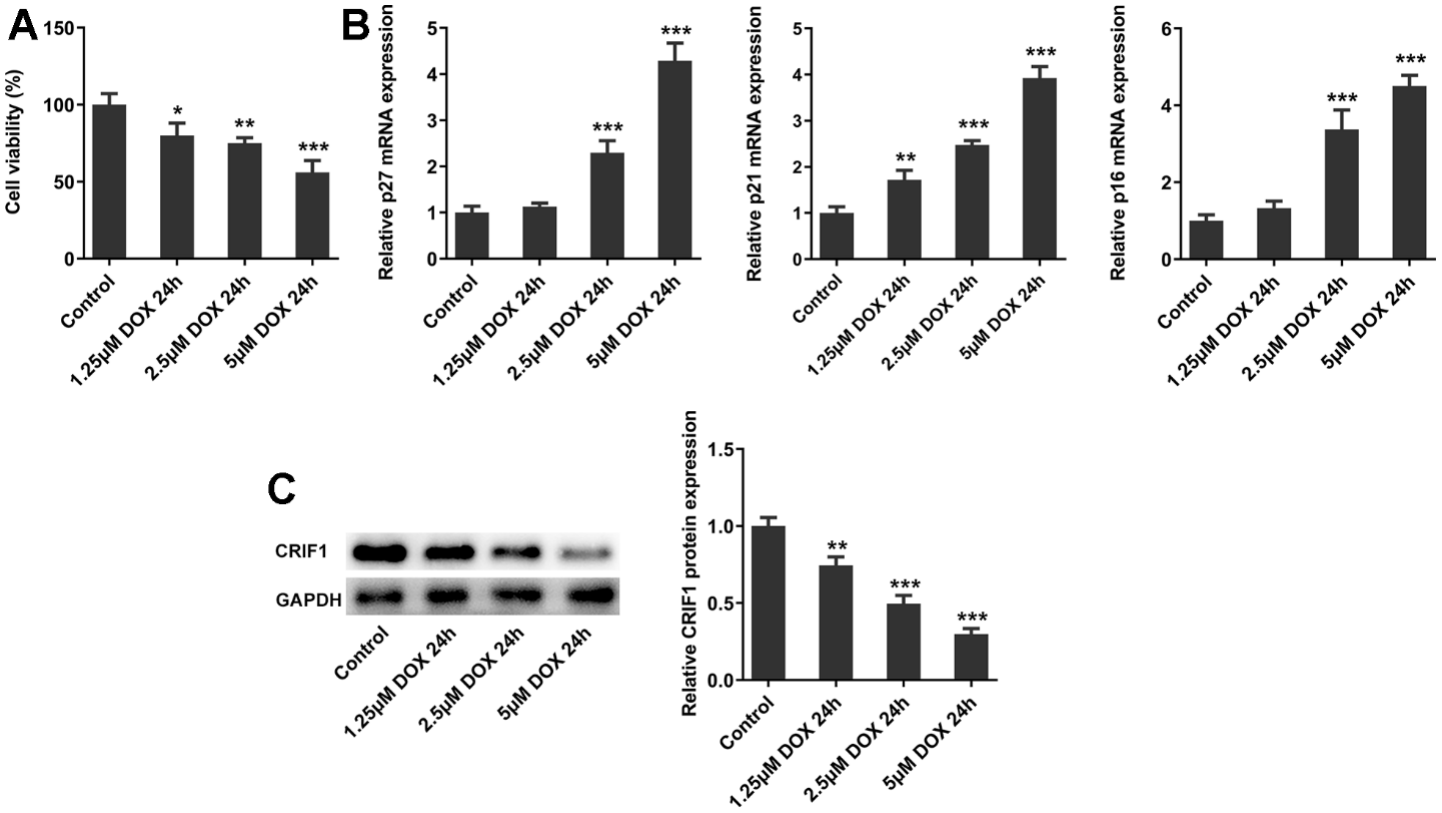
**The expression of CRIF1 was decreased in DOX-induced AC16 cells.** (**A**) The cell activity was detected by CCK8. (**B**) RT-qPCR detected the expression of p27, p21, and p16 in AC16 cells. (**C**) The protein expression of CRIF1 was examined by western blot. *P<0.05, **P<0.01, ***P<0.001 vs Control.

Subsequently, we further explored the molecular mechanism in cell experiments. AC16 cells were induced by DOX at different concentrations, and the cell activity was decreased significantly with the increasing DOX concentrations (Figure 5A). In addition, after DOX induction, the mRNA expressions of p27, p21 and p16 in cells were significantly increased (Figure 5B). Western blot results showed that with the increasing DOX concentrations, the expression of CRIF1 in cells decreased in a concentration-dependent manner (Figure 5C). The results of cell experiments were consistent with those of animal experiments.

### CRIF1 overexpression inhibited oxidative stress and senescence in DOX-induced AC16 cells

**Figure 6:**
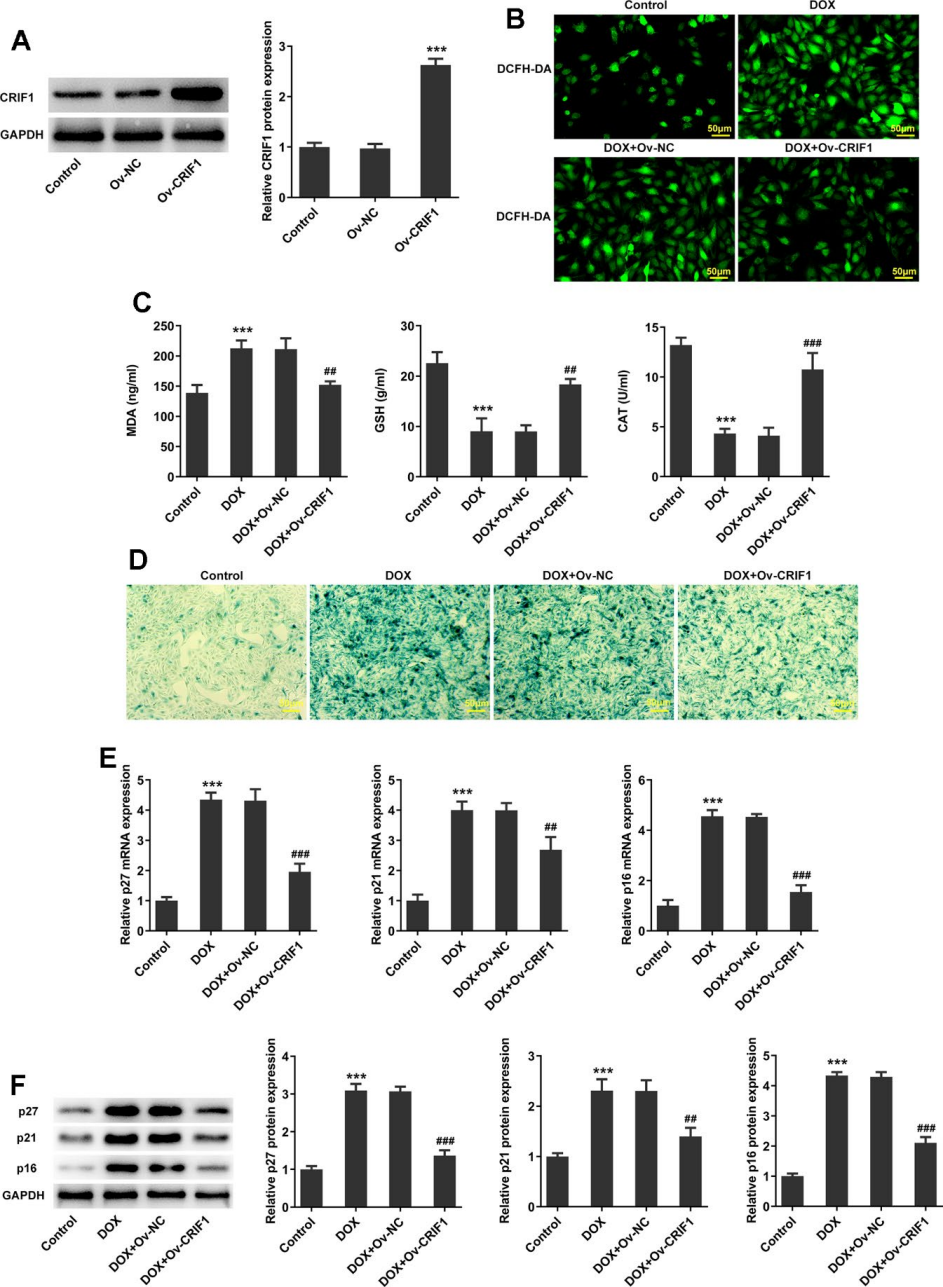
**CRIF1 overexpression inhibited oxidative stress and senescence in DOX-induced AC16 cells.** (**A**) AC16 cells were transfected with Ov-NC or Ov-CRIF1, and the overexpression efficacy of CRIF1 was detected by western blot. ***P<0.001 vs Ov-NC. (**B**) DCFH-DA staining was used to detect ROS levels. (**C**) The expression levels of MDA, GSH and CAT in cells were detected by biochemical kits. (**D**) SA-β-gal staining was used to observe the level of cell senescence. (**E**, **F**) RT-qPCR and western blot were used to detect the expression of p27, p21, and p16. ***P<0.001 vs Control; ##P<0.01, ###P<0.001 vs DOX+Ov-NC.

To explore the regulation of CRIF1 *in vitro*, AC16 cells were transfected with Ov-NC or Ov-CRIF1, and the results showed that compared to the Ov-NC group, the expression level of CRIF1 was significantly elevated in Ov-CRIF1 group (Figure 6A). Next, AC16 cells with or without CRIF1 overexpression were treated with 5 μM of DOX for 24 h. It was found from Figure 6B, 6C that compared with the Control, DOX greatly promoted ROS production and MDA level, and reduced GSH and CAT activities, which was partly abolished by CRIF1 overexpression, suggesting that CRIF1 overexpression could attenuate DOX-induced oxidative stress in AC16 cells. SA-β-gal staining was used to observe the level of cell senescence, and the results showed that compared with DOX+Ov-NC group, cell senescence in DOX+Ov-CRIF1 group was alleviated (Figure 6D). Meanwhile, DOX-caused high expression of p27, p21 and p16 was partly inhibited by overexpression of CRIF1 (Figure 6E, 6F).

### CRIF1 overexpression inhibited PXDN expression in DOX-induced myocardial tissue and AC16 cells

**Figure 7:**
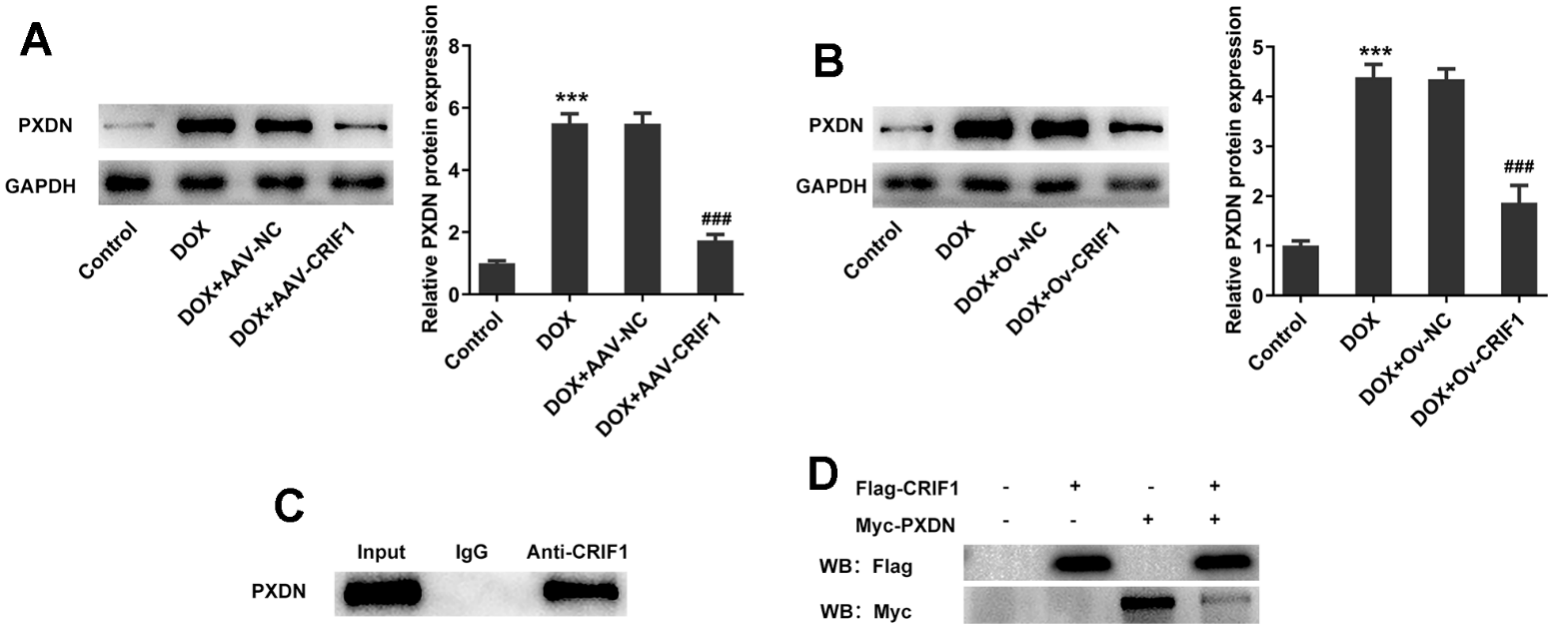
**CRIF1 overexpression inhibited PXDN expression in DOX-induced myocardial tissues and AC16 cells.** (**A**) Western blot detected the expression of PXDN in myocardial tissues. (**B**) Western blot detected the expression of PXDN in AC16 cells. (**C**) CO-IP experiment was used to detect the relationship between CRIF1 and PXDN. (**D**) 293T were transfected with flag-CRIF1 and/or myc-PXDN, and CO-IP assay followed by western blot was performed to examine the myc-tagged protein or flag-tagged protein. ***P<0.001 vs Control; ###P<0.001 vs DOX+Ov-NC.

In animal experiments, we found that the expression of PXDN was significantly increased after DOX induction, while the expression of PXDN was significantly decreased after further overexpression of CRIF1 in the myocardial tissues of mice (Figure 7A). The trend was also consistent in cells (Figure 7B). CO-IP experiment results showed that CRIF1 could interact with PXDN (Figure 7C). Further, to further validate the interaction, 293T were transfected with flag-CRIF1 and/or myc-PXDN, and CO-IP assay followed by western blot was performed to examine the myc-tagged protein or flag-tagged protein. The results showed that protein expression of myc-PXDN was markedly inhibited in flag-CRIF1-mediated immunoprecipitants (Figure 7D). The above data suggested that CRIF1 could bind to PXDN and inhibit PXDN expression.

### CRIF1 overexpression alleviated oxidative stress and cell senescence by inhibiting PXDN in DOX-induced AC16 cells

**Figure 8:**
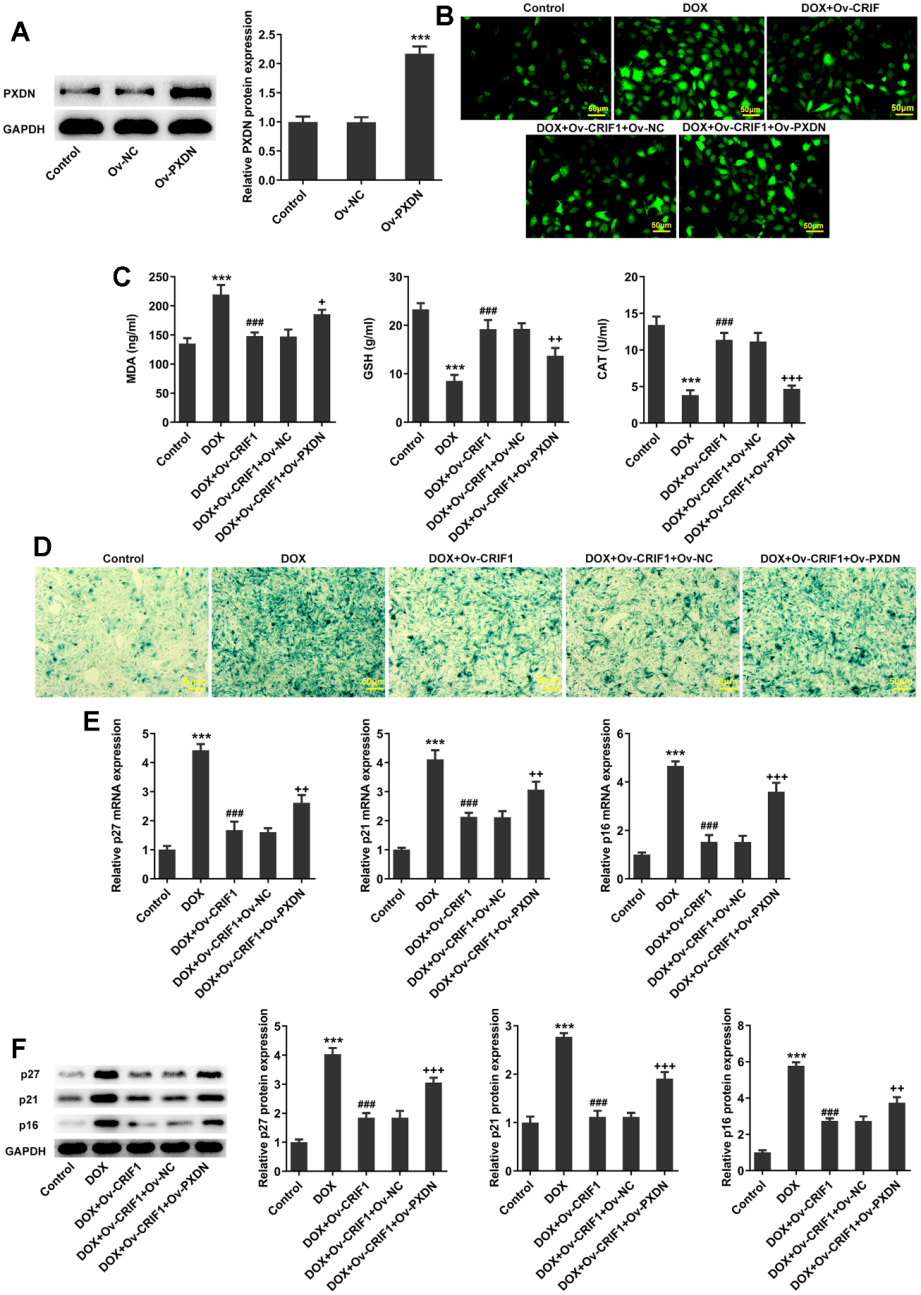
**CRIF1 overexpression alleviated oxidative stress and cell senescence by inhibiting PXDN in DOX-induced AC16 cells.** (**A**) AC16 cells were transfected with Ov-NC or Ov-PXDN, and the overexpression efficacy of PXDN was detected by western blot. ***P<0.001 vs Ov-NC. (**B**) DCFH-DA staining was used to detect ROS levels. (**C**) The expression levels of MDA, GSH and CAT in cells were detected by biochemical kits. (**D**) SA-β-gal staining was used to observe the level of cell senescence. (**E**, **F**) RT-qPCR and western blot detected the expression of p27, p21, and p16. ***P<0.001 vs Control; ###P<0.001 vs DOX; +P<0.05, ++P<0.01, +++P<0.001 vs DOX+Ov-CRIF1+Ov-NC.

AC16 cells were transfected with Ov-PXDN to over-express PXDN (Figure 8A). Subsequently, AC16 cells were transfected with Ov-CRIF1 alone or co-transfected with Ov-CRIF1 and Ov-NC/Ov-PXDN, followed by DOX induction. It was noticed that ROS and MDA levels increased, while GSH and CAT activities decreased in DOX+Ov-CRIF1+Ov-PXDN group relative to the DOX+Ov-CRIF1+Ov-NC group (Figure 8B, 8C). SA-β-gal staining showed that overexpression of PXDN could reverse the inhibitory effect of CRIF1 overexpression on cell senescence (Figure 8D). It was also found that overexpression of PXDN could reverse the inhibitory effect of CRIF1 overexpression on p27, p21 and p16 expressions (Figure 8E, 8F).

## DISCUSSION

Myocardial senescence is closely related to the occurrence and development of cardiovascular diseases. Studying the biological characteristics of myocardial senescence is of great significance for delaying or even reversing cardiomyocyte senescence [15]. In this paper, we discussed the important role of CRIF1 in myocardial senescence, so as to clarify the pathogenesis of myocardial senescence and provide a theoretical basis for the clinical treatment of cardiovascular diseases.

In our experiment, it was found that the expression of CRIF1 was significantly decreased in DOX-induced mice and cardiomyocytes. CRIF1 is a multifunctional protein widely expressed in the nucleus and cytoplasm that is involved in the construction of the large subunit of mitochondrial ribosomes. CRIF1 can also induce cell cycle arrest, regulate cellular oxidative stress and transcriptional activity of transcription factors, as well as regulate various physiological functions of cells [16]. A previous study has shown that CRIF1 can interact with Gadd45 (Growth arrest and DNA-damage inducible 45) family proteins to regulate cell cycle [17]. Increased CRIF1 expression leads to cell cycle arrest [18]. Also, after the cell division reaches a certain number of times, CRIF1 will stop the division and proliferation, and cannot enter the new cell cycle again, and the expression levels of senescence markers are significantly increased [19]. Existing study has shown that CRIF1 deficiency induces endothelial cell senescence [20], indicating that CRIF1 can participate in the biological process of senescence. Therefore, it is reasonable to assume that CRIF1 plays an important role in the process of myocardial senescence. In our experiment, it was found that overexpression of CRIF1 could significantly improve oxidative stress damage and senescence of mouse myocardial tissues and cardiomyocytes induced by DOX. In terms of oxidative stress, it has been shown that CRIF1 deficiency induces P66SHC-mediated oxidative stress and endothelial activation [21]. Co-activation of PKC-δ by CRIF1 regulates oxidative stress in bone marrow pluripotent mesenchymal stromal cells after irradiation by phosphorylation of NRF2 Ser40 [22]. The above research results were consistent with the results of our article.

Moreover, we found that overexpression of CRIF1 could attenuate mitochondrial dysfunction of myocardial tissues in senescent mice. Senescence can lead to cardiac dysfunction, decreased myocardial contractility, fibrosis of myocardial tissues, inflammation, oxidative stress and many other pathological manifestations [23]. Mitochondria are the power of cells, and are dynamic organelles that cycle between fusion and division states. Previous study has believed that mitochondrial dysfunction is the core of the senescence theory, and senescence is related to the decline of mitochondrial function [24]. In addition, myocardial mitochondrial fusion decreases and division increases during senescence, resulting in decreased MMP, calcium overload and changes in mitochondrial enzyme activity, thus affecting myocardial energy metabolism [10]. In addition, inhibition of the mitochondrial protein CRIF1 interferes with mitochondrial function, depolarizes membrane potential, and increases ROS levels in endothelial cells [25]. Consistently, our experimental results presented that myocardial senescence occurred in DOX-caused mice or cardiomyocytes, and CRIF1 overexpression greatly inhibited the DOX-induced oxidative stress, mitochondrial dysfunction and myocardial senescence *in vivo* and *in vitro*.

Next, we further explored the regulatory mechanism of CRIF1 at the cellular level. It was predicted by Monarch Initiative (https://monarchinitiative.org/) that CRIF1 could combine with PXDN, which was then verified by CO-IP assay. Meanwhile, we also found that CRIF1 inhibited PXDN expression. PXDN, also known as vascular peroxidase 1 (VPO1), is a member of the peroxidase family that exacerbates oxidative stress by producing hypochlorous acid (HOCl) [26]. It has been suggested that PXDN expression increases during myocardial ischemia-reperfusion injury and is involved in promoting myocardial fibrosis [27]. Study has shown that palmitic acid can induce increased expression of PXDN in insulin-resistant cardiomyocytes [28]. At the same time, the expression of PXDN is increased in endothelial cells induced by high glucose, and the interference of PXDN inhibits endothelial cell senescence [29]. PXDN is involved in oxidative stress and senescence, but whether PXDN is involved in regulating oxidative stress and senescence induced by DOX remains unclear. In our experiment, we found that the expression of PXDN was significantly increased in DOX-induced mouse myocardial tissues and cardiomyocytes. Overexpression of PXDN could reverse the ameliorating effect of CRIF1 overexpression on oxidative stress and senescence of DOX-induced cardiomyocytes.

In conclusion, we found that CRIF1 inhibited PXDN to regulate mitochondrial function, thereby improving myocardial senescence induced by DOX. Our paper might provide a reliable theoretical basis for the clinical treatment of myocardial senescence.
